# Autoimmune Metaplastic Atrophic Gastritis Reporting: Are Pathologists and Endoscopists on the Same Page?

**DOI:** 10.3390/diagnostics15222906

**Published:** 2025-11-17

**Authors:** Nicole Vienneau, Hwajeong Lee, Xulang Zhang, Eundong Park, Madeline Cleary, Jing Zhou, Shunsa Tarar, Meng Liu, Micheal Tadros

**Affiliations:** 1 Department of Pathology and Laboratory Medicine, Albany Medical Center, 43 New Scotland Avenue, Albany, NY 12208, USA; 2 Gastroenterology, Department of Medicine, Albany Medical Center, 43 New Scotland Avenue, Albany, NY 12208, USA; 3 Department of Medicine, Albany Medical Center, 43 New Scotland Avenue, Albany, NY 12208, USA

**Keywords:** AMAG, autoimmune metaplastic atrophic gastritis, endoscopy, histology, neuroendocrine tumor

## Abstract

**Background/Objectives:** Autoimmune metaplastic atrophic gastritis (AMAG) is a chronic, autoimmune-mediated condition associated with increased risk of malignancy and nutritional deficiencies, yet diagnostic and follow-up processes remain inconsistent and unclear. This study investigates follow-up testing performance in patients with AMAG and neuroendocrine tumors (NET), as well as the correlation between endoscopic impressions and histologic findings. **Methods:** We retrospectively analyzed 65 gastric biopsies with final diagnoses or comments mentioning the possibility of AMAG, 12 of which included well-differentiated WHO grade 1 NET arising in AMAG. H&E slides were reviewed to assess atrophy severity, the presence or absence of enterochromaffin-like (ECL) cell hyperplasia, and Helicobacter organisms. The final diagnostic line or comments made were scored from 1 to 5, based on the strength of the language used to alert the treating clinician to the likelihood of AMAG. Corresponding endoscopy reports were scored from 1 to 5 based on the likelihood of the reports documenting AMAG features. Data regarding follow-up laboratory testing relevant to AMAG and biopsy performance were collected from the electronic medical records. **Results:** Endoscopy scores showed no significant associations with the histology comment score or atrophy grade. The histology comment score was positively associated with performing at least a total of three laboratory tests (*p* = 0.03). No association was found between the presence or absence of follow-up biopsy and histology comment score (*p* = 0.60). Follow-up biopsy was more common in patients with NET than those with AMAG without NET (*p* < 0.001). **Conclusions:** Poor endoscopic–histologic correlation with variable follow-up practices highlights the need for standardized protocols in AMAG management. Enhanced adherence to biopsy guidelines, standardized pathology reporting, and consistent surveillance, particularly for patients with AMAG without NET, are imperative to improve diagnosis and outcomes. Future research should focus on optimizing endoscopic techniques, standardizing serological tests, and establishing evidence-based surveillance protocols for AMAG patients.

## 1. Introduction

Autoimmune metaplastic atrophic gastritis (AMAG) is an autoimmune-mediated condition resulting in the destruction of parietal cells in the fundal and corporal gastric mucosa, sparing the antrum [1]. Possible mechanisms for AMAG include the formation of antibodies directly against parietal cells and intrinsic factor. Alternatively, the molecular similarity between the beta subunit of *H. pylori* urease and the beta unit of ATPase on parietal cells can cause parietal cells’ destruction following Helicobacter gastritis [2].

Histologically, the consequence of this chronic inflammatory process includes loss of normal gastric mucosa that is replaced by fibrous tissue (nonmetaplastic atrophy) or intestinal-type epithelium, pyloric-type epithelium, or Paneth cells (metaplastic atrophy). Furthermore, a reduction in hydrochloric acid (HCl) production from diminished parietal cells stimulates gastrin overproduction from antral G-cells, resulting in enterochromaffin-like (ECL) cell hyperplasia in the gastric corpus. Over time, the lack of intrinsic factor can result in the inability to absorb vitamin B12, resulting in macrocytic anemia (pernicious anemia) [3]. Knowledge of the pathologic physiological changes in these patients can direct serological testing to aid in diagnosis, including antibodies against parietal cells and intrinsic factor, serum levels of vitamin B12, chloride, gastrin, and pepsinogen I/II ratio (decreased due to loss of chief cells), and anemia testing [3,4]. Also, metaplastic atrophy and ECL cell hyperplasia significantly increase the risk of gastric adenocarcinoma and type 1 neuroendocrine tumors (NET), respectively [5,6].

Obtaining gastric biopsies via upper gastrointestinal endoscopy is the first step in diagnosing AMAG. However, endoscopic impressions of gastric atrophy may be prone to low sensitivity and specificity, and high inter-observer variation [7,8]. Gastric mucosa may not display any significant changes in early stages of atrophy, and atrophy located in the oxyntic mucosa alone may not be prominent [1]. In 1990, a group of pathologists and gastroenterologists created the Sydney System, an etiology- and location-based classification system of gastritis, with the updated version created in 1994 adopted as the standard worldwide [9]. This system proposes that adequate, mapped sampling is essential to accurately characterize gastritis, especially AMAG. It recommends that biopsies are received in separate, labeled containers by location, with at least two each from the corpus and antrum, and with one from the angularis mucosa. The Operative Link for Gastritis Assessment (OLGA) builds upon this framework, providing a staging system for gastric mucosal atrophy in terms of gastric cancer risk by combining the extent of histologic atrophy with the topographical location [10]. However, improper biopsy sampling, including unspecified gastric locations, negatively impacts the pathologist’s ability to diagnose AMAG and utilize these reporting systems. In one study of gastric biopsy sets from 379,667 patients, only 6.8% of the specimens were delivered in two separate containers containing tissue from both the corpus and antrum [11].

Current United States (US) guidelines lack clarity regarding endoscopic sampling, pathology reporting, and patient surveillance in AMAG [12]. A non-standardized approach in the communication between gastroenterologists and pathologists calls into question the ability to achieve accurate clinicopathologic correlation in this disease. In our study we evaluated what follow-up was performed, if any, when a pathologist raised the likelihood of AMAG in a gastric biopsy. Secondly, we aimed to correlate the accuracy of concurrent endoscopic impressions with histopathological findings. We hypothesize that patients with stronger comments raising the possibility of AMAG in their pathology reports or more severe histopathologic changes in AMAG are followed more closely with more tests. Likewise, in retrospect, endoscopic mucosal abnormalities might have been more conspicuous in these patients compared to those with weaker comments in their pathology reports or subtle/mild histopathologic changes. We aim to provide foundational histopathologic and endoscopic data that can be used to devise a practical endoscopic scoring and histologic reporting system for AMAG in the future.

## 2. Materials and Methods

### 2.1. Case Selection

Pathology database (2013–2023) was searched for gastric biopsies with final diagnoses or comments mentioning the possibility of AMAG or showing well-differentiated neuroendocrine tumor (NET). Corresponding hematoxylin and eosin (H&E) stained slides and available follow-up biopsies were reviewed and cases that were unlikely to represent AMAG or type 1 NET (NET arising in atrophic gastritis) were excluded. In other words, biopsies included demonstrated atrophy, metaplasia, inflammation, and ECL cell hyperplasia of corpus-type mucosa, and relative sparing of the antral mucosa in the initial biopsy and/or follow-up biopsy [13]. Finally, 65 cases were included in the study, in which 12 showed well-differentiated WHO grade 1 NET arising in AMAG.

### 2.2. Biopsy Review

The H&E slides were reviewed to assess the degree of mucosal atrophy (grade 0–3; no atrophy (grade 0), 1–30% atrophy (grade 1), 31–60% atrophy (grade 2), and >60% atrophy (grade 3)) using a previously published gastric atrophy grading scheme [9]. When more than one biopsy was available and the fragments showed different atrophy grades, the mean of the atrophy grades was recorded. Available immunohistochemistry slides were reviewed along with H&E for the presence or absence of ECL cell hyperplasia/NET [14]. and *Helicobacter* organisms. The ECL cell hyperplasia pattern was scored as follows: 0 for no hyperplasia, 1 for linear hyperplasia, 2 for micronodular hyperplasia, and 3 for the presence of NET. If multiple patterns were observed, the highest score was assigned.

For targeted biopsies (see below), the H&E slides were reviewed to reconcile endoscopic abnormality and histologic features.

### 2.3. Histology Comment Score

**Figure 1:**
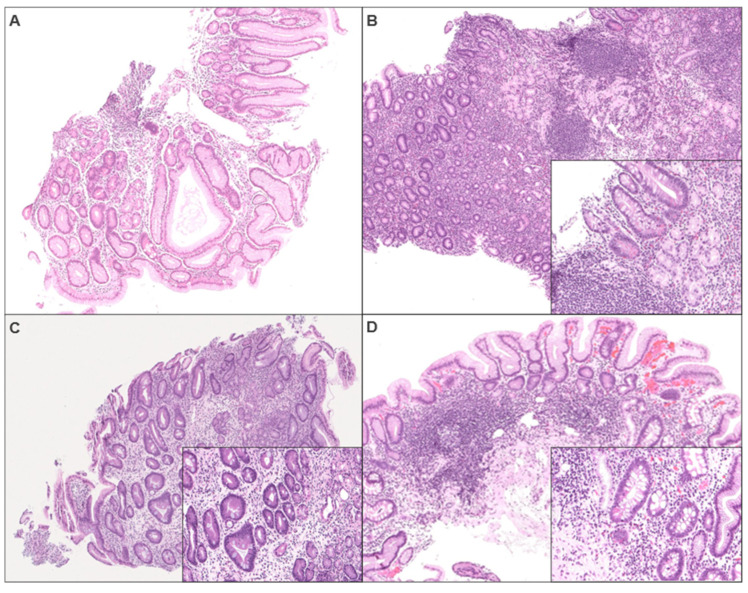
(H&E 5×, 20× (inset)). Pathology report score 1: polypoid pseudopyloric metaplasia vs. hyperplastic polyp (**A**). Score 2: *H. pylori* positive gastric corpus biopsy with chronic active gastritis, intestinal metaplasia, Paneth cell metaplasia, and atrophy (**B**). Score 3: *H. pylori* negative random gastric biopsy demonstrating chronic active gastritis with atrophy, intestinal metaplasia, and linear ECL cell hyperplasia (**C**). Score 4/5: gastric corpus biopsy demonstrating chronic atrophic gastritis with ECL cell hyperplasia and intestinal, pseudopyloric, and Paneth cell metaplasia (**D**).

The final diagnostic line or comments made by original pathologists were scored from 1 to 5, based on the strengths of the language used to alert the clinician of the likelihood of AMAG by a GI pathologist (H.L.) (Table 1, Figure 1).

### 2.4. Endoscopy Report Score

**Figure 2:**
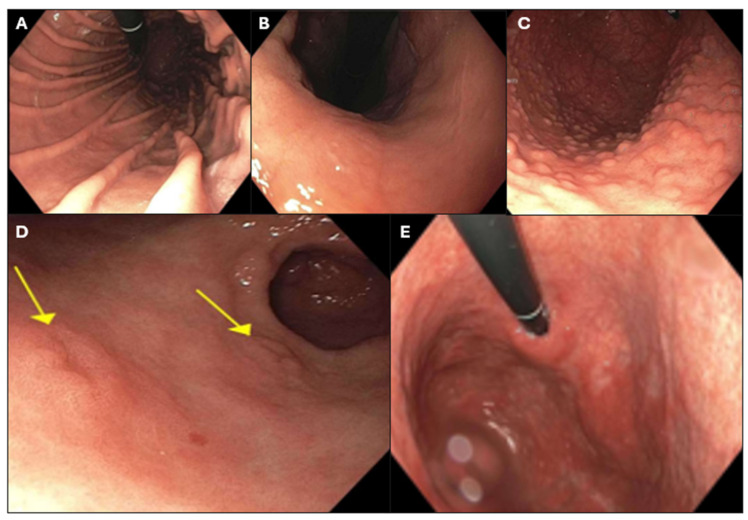
Endoscopy score 1: normal gastric corpus (**A**). Score 2: gastric erythema (**B**). Score 3: cobblestoning with polypoid-appearing tissue (**C**). Score 4: gastric corpus with yellow arrows indicating ulcerative lesions (**D**). Score 5: gastric corpus atrophy (**E**).

Corresponding endoscopy reports were available for 63 cases, which were retrieved and deidentified. Twenty-nine reports contained representative endoscopic images. The reports were scored from 1 to 5 based on the likelihood of the endoscopic reports documenting AMAG features (Table 2, Figure 2), by (1) a pathology research associate (X.Z.) (ES1) and (2) reinterpreted and scored by a gastroenterology trainee and attending gastroenterologist (M.C. and M.T.) (ES2). Gastroenterology raters reviewed endoscopic images when available. Indications for biopsy and biopsy site (antrum, corpus, cardia, greater curvature, lesser curvature, and targeted biopsy of a lesion) were recorded.

### 2.5. Chart Review

Data regarding demography, follow-up duration, other autoimmune diseases, laboratory testing relevant to AMAG to include *H. pylori* serology testing, serum hypochlorhydria, anti-parietal cell antibody, vitamin B12, hemoglobin, serum gastrin, and pepsinogen I/II were collected from the electronic medical records. The number of follow-up laboratory tests was defined as the total count of the aforementioned tests (max. 7).

### 2.6. Statistical Analyses

Statistical analyses were performed using R Studio version 4.3.3. Descriptive statistics for the cohort were presented using the mean, median, and range for continuous and ordinal categorical variables, and frequency and percentage for dichotomous categorical variables. Hypothesis testing was conducted using Fisher’s exact test for dichotomous variables, the Wilcoxon rank-sum test for comparisons between dichotomous and ordinal variables, and the Spearman correlation for ordinal variables. For correlation analyses between histologic and endoscopic findings, *p*-value adjusted by false discovery rate (FDR) was used. Statistical significance was defined as *p* < 0.05.

## 3. Results

### 3.1. Cases

Sixty-five patients were identified. Descriptive statistics of the cohort are outlined in Table 3. Fifty-three patients had final diagnoses or comments mentioning the possibility of AMAG in their pathology reports. Twelve patients had well-differentiated NET at the time of the biopsy, among which the background flat mucosa was assessed with relevant comments in 10 (83%) cases. Thus, the possibility of AMAG was addressed in 63 (97%) cases in the initial pathology report. The male to female ratio was 1:1.6, and the mean age was 64.6 (range 30–86) years. Thirteen patients had conditions related to immune dysregulation including hypothyroidism (*n* = 7), type 1 diabetes (*n* = 2), Grave’s disease (*n* = 1), Hashimoto’s thyroiditis (*n* = 1), and other (*n* = 4; unspecified thyroid disease, IgA nephropathy, ANA positivity, anti-intrinsic factor antibody positivity, Crohn’s disease, etc.).

### 3.2. Indication for Endoscopy and Tissue Sampling

The indication for esophagogastroduodenoscopy (EGD) were anemia/GI bleed (*n* = 19), dysphagia /pain (*n* = 27), follow-up for known gastric pathology/neoplasm (*n* = 18), follow-up for known esophageal pathology including reflux (*n* = 8), other GI symptoms (nausea, diarrhea, weight loss, etc.) (*n* = 8), and other (pancreatobiliary tract evaluation, imaging abnormality, family history, pre-operative evaluation, etc.) (*n* = 5) (Table 3). At the time of initial EGD procedure, flat mucosa, including the antrum (*n* = 31), corpus (*n* = 30), cardia (*n* = 4), lesser curvature (*n* = 1), and random mucosa (*n* = 21), were biopsied in 54 (83%) patients, with additional targeted biopsy of a lesion being performed in 21 (32%) patients. In 11 (17%) patients, targeted biopsy (of polyp, nodule, ulcer, etc.) was performed, without evaluation of flat mucosa (Table 3). Targeting a lesion without sampling of adjacent flat mucosa was more common when a patient presented with NET compared to AMAG without NET (6 of 12 (50%) vs. 5 of 53 (9%); *p* = 0.003). In 24 (37%) patients, the antrum and corpus were labeled and sampled in separate containers.

### 3.3. Biopsy Review

The biopsies showing features of potential AMAG or NET were from the antrum (*n* = 2), corpus (*n* = 46), cardia (*n* = 5), greater curvature (*n* = 2), lesser curvature (*n* = 1), and random mucosa (*n* = 19). Among them, 29 were targeted biopsies of a lesion (Table 3).

Atrophy grade was assigned for all 65 cases. The mean and median atrophy grades were 2.8 and 3, respectively, with grade 3 being the most common (54 of 65; 83%). Synaptophysin or chromogranin immunohistochemistry to evaluate ECL cell hyperplasia/NET was performed on 61 (94%) cases at the time of initial diagnostic workup. Based on immunohistochemistry and H&E stain, 1 case was deemed to show no ECL cell hyperplasia, 5 showed linear, 48 showed linear and micronodular ECL cell hyperplasia, and 12 cases showed NET. Immunohistochemistry for *Helicobacter* organisms had been performed on 59 cases at the time of initial diagnostic workup and 56 (95%) were negative for organisms (Table 3).

Next, targeted biopsies were reviewed to reconcile endoscopic abnormalities and pathologic features. The most common endoscopic abnormality that was targeted and sampled was polyp/nodule/mass (*n* = 20), followed by ulcer/erosion (*n* = 6), scar (*n* = 2), pigmented spot (*n* = 1), tattoo (*n* = 1), and previous procedure site (*n* = 1). Eight (40%) of the polyp/nodule/mass lesions were NET and the remainder showed a spectrum of AMAG including pseudopolyp (relative elevation of non-atrophic mucosa surrounded by atrophic mucosa), foveolar hyperplasia and florid pseudopyloric metaplasia. Two (33%) of six erosion/ulcers were NET, and the remainder showed atrophy. One scar lesion showed atrophy and the other showed xanthoma. One pigmented spot showed NET and the tattoo site biopsy showed atrophy without pigments. In summary, 38% (11 of 29) of targeted biopsies showed NET, and the tumors presented as polyp, nodule, mass, erosion, and pigmented mucosa. However, atrophy-related changes also presented as a polyp/nodule, ulcer/erosion, and scar.

Follow-up biopsies (2014–2024) were available in 24 patients, and the mean number of follow-up biopsies (EGD procedure) was 2.2 (range 1–8). The presence of AMAG was confirmed by follow-up biopsies for two NET cases that did not show sufficient background flat mucosa for evaluation at the time of initial biopsy.

### 3.4. Histology Comment Scores and Their Correlation with Histology

A histology comment score was assigned for 63 cases. In two NET cases, no comment was made regarding the possibility of AMAG. The mean and median histology comment score was 4.2 and 5, respectively, with score 5 (39 of 63; 57%) being the most common, followed by score 3 (*n* = 11). No statistically significant associations were found between the histology comment score and ECL cell hyperplasia (r_s_ = 0.05, *p* = 0.69) or atrophy grade (r_s_ = 0.15, *p* = 0.24), while there was a statistically significant, positive correlation between the ECL cell hyperplasia and atrophy grade (r_s_ = 0.32, *p* = 0.01; Table 4).

### 3.5. Endoscopy Report Scores and Their Correlation with Histology

Endoscopy reports were available in 63 cases. The reports were initially scored (ES1) by a pathology research associate (by X.Z., with no training in gastroenterology) based on the terminology used in the reports. Next, the reports, along with available endoscopic images, were reinterpreted and scored (ES2) by a gastroenterology trainee (M.C.) and an attending gastroenterologist (M.T.). ES1 and ES2 were strongly correlated (r_s_ = 0.78, *p* < 0.01; Table 4). Likewise, score 3 (*n* = 24 and 20, respectively) was the most common, followed by score 2 (*n* = 14 and 16, respectively) in both ES1 and ES2 (Table 3).

No statistically significant association was found between ES1 and ECL cell hyperplasia (r_s_ = 0.30, *p* = 0.06) histology comment score (r_s_ = 0.09, *p* = 0.72) and atrophy grade (r_s_ = 0.04, *p* = 0.75; Table 4). Likewise, no statistically significant associations were found between ES2 and ECL cell hyperplasia (r_s_ = 0.14, *p* = 0.52), histology comment score (r_s_ = 0.13, *p* = 0.54), and atrophy grade (r_s_ = 0.05, *p* = 0.75; Table 4).

### 3.6. Histology Comment Score and Endoscopy Report Score vs. Tissue Sampling and Gender

We hypothesized that histology comment score and/or endoscopy report score may be influenced by tissue sampling and the patient’s gender. When the cases were divided according to tissue sampling (separate sampling of antrum and corpus (*n* = 24) vs. random and or targeted biopsy (*n* = 41)), there was no difference in the histology comment scores in the two subgroups. Interestingly, the mean of ES1 was higher when the antrum and corpus were separately sampled and labeled (*p* = 0.03). Similarly, the mean of ES2 was marginally higher when the samples were separately submitted from the antrum and corpus than when random or targeted biopsies were performed (*p* = 0.06). A similar trend was observed when the cases were divided by gender. While no difference was noted in the histology comment score between male and female patients, ES1 was marginally higher and ES2 was higher in female patients than males (*p* = 0.05 and *p* = 0.01, respectively). There was no association between gender and tissue sampling.

### 3.7. Follow-Up

Follow-up was available in 26 (40%) patients with the mean and median follow-up duration of 29.4 and 26.3 months (range 2–84 months), respectively.

#### 3.7.1. Follow-Up Laboratory Testing

Hemoglobin testing was the most common laboratory test performed (*n* = 54), with serum pepsinogen I/II never performed (*n* = 0; Table 5). Out of seven laboratory tests that are relevant to AMAG, at least one test was performed in 57 (88%) patients. The mean number of tests was 2.4 (range 0–5). The number of tests was not associated with histology comment score (*p* = 0.08, r_s_ = 0.23), ES1 (*p* = 0.48, r_s_ = 0.09), or ES2 (*p* = 0.22, r_s_ = 0.16). However, when the number of tests was divided into low (0–2 tests) vs. high (3–7 tests), the histology comment score was positively associated with performing a high number of tests, compared to low (*p* = 0.03).

#### 3.7.2. Follow-Up Biopsy vs. Histology and Histology Comment Score

When the patients were divided into those with (*n* = 24) and without (*n* = 41) follow-up biopsies, the subgroup with follow-up biopsy showed more advanced ECL cell hyperplasia (*p* < 0.001) and tended to show higher grades of atrophy (*p* = 0.13) compared to the latter subgroup. Similarly, follow-up biopsy was more common when patients presented with NET (10 of 12) than AMAG without NET (14 of 53) (*p* < 0.001). However, no association was found between the presence or absence of follow-up biopsy and histology comment score (*p* = 0.62).

#### 3.7.3. Follow-Up Biopsy vs. Endoscopy Report Score

When the patients were divided into those with (*n* = 24) and without (*n* = 41) follow-up biopsies, the former subgroup tended to have a higher ES1 score (*p* = 0.06) than the latter. No such trend was observed between the presence or absence of follow-up biopsy and ES2 (*p* = 0.60).

## 4. Discussion

Despite the pathophysiology of AMAG being generally well-defined, factors relating to disease aggressiveness and rate of progression are not well known, making clinical trajectories difficult to predict. As such, optimal endoscopic surveillance intervals with or without biopsy are currently unclear. The American Gastroenterological Association (AGA) recommends that the treating clinician decide the surveillance plans [12]. The American Society for Gastrointestinal Endoscopy does not offer any recommendation for AMAG management, only suggesting that endoscopy be performed within 6 months of pernicious anemia diagnosis [15]. We retrospectively collected gastric biopsies from 65 patients who had a pathological diagnosis of probable AMAG and/or NET, assessed AMAG histopathologic features, and correlated these with endoscopic findings. We also investigated whether a follow-up biopsy was performed following pathologic diagnosis, as well as whether relevant laboratory testing was conducted. Our results support the need for the development of uniform clinical follow-up for patients with AMAG. We found that whether a follow-up biopsy was performed or not had no association with the histology comment score. However, follow-up biopsies were more commonly performed when the patient presented with NET, had more advanced ECL cell hyperplasia, or had more severe atrophy. Histology comment score was correlated with performing greater than three laboratory tests.

Adequate histologic assessment of AMAG first requires proper topographic-based biopsy sampling for the pathologist to make a diagnosis, and our findings support the literature that this is poorly adhered to in the average practice [11,16]. We observed that only 40% of cases included separate sampling of the antrum and corpus. In addition to recommendations from the Sydney system [9] and OLGA [10], the AGA does advise obtaining separately labeled biopsies from the corpus and antrum/incisura, at minimum, when endoscopic features of atrophic gastritis are present [12]. Lack of adherence may reflect the non-specific endoscopic findings of AMAG, lack of awareness, or absence of implemented protocols.

Furthermore, we noticed that the sampling of flat mucosa surrounding a lesion was less frequently performed for patients with NET compared to those without. The sampling of mucosa surrounding a lesion is of particular importance for gastric NETs, as histology will aid in classifying the tumor and has implications for prognosis: type I NETs (due to chronic atrophic gastritis) have a low risk of metastases with normal life expectancy, while type III NETs (sporadic; histologically normal surrounding mucosa) have a high risk of metastases with overall poor prognosis [17]. The European Neuroendocrine Tumor Society (ENETS) also recommends separate sampling of the antral and corporal mucosa in the evaluation of gastric NETs, to allow for proper patient management [18]. A previous study has found that, when separate containers with at least two antral and two corporal specimens (Sydney system compliant) were submitted for histopathological analysis, the diagnostic yield was increased in the evaluation of intestinal metaplasia compared to fewer specimens or unspecified sites [11]. Presumably, this would translate to other gastric conditions that require adequate representation of the stomach, including AMAG.

Although the histological patterns of AMAG are well-characterized, no single histological feature is diagnostic. The early phases of AMAG often present quite subtly with non-specific changes, including diffuse chronic inflammatory cell infiltrates, parietal cell pseudohypertrophy, and prominent eosinophils in the lamina propria [9,19], potentially resulting in the pathologist overlooking the diagnosis. Most of our cases showed severe atrophy (83%) with ECL cell hyperplasia present (82%), suggesting that these features may reliably prompt pathologists to consider AMAG. Even so, the strength of the language used in offering AMAG as a diagnosis was not significantly associated with any histologic feature, tissue sampling intention, or patient sex. This may indicate that the histology comment strength is related to a pathologist’s confidence level and experience. In the case of less experienced pathologists or areas serving populations with a high AMAG prevalence, a gastrin immunohistochemical stain may be helpful to confirm the absence of gastrin cells from true corpus mucosa [19]. At our institution, our study highlights that additional work-up to evaluate for *H. pylori* (the major contributor/cause for environmental atrophic gastritis [9,20]) and ECL cell hyperplasia (supportive of AMAG) is routinely performed when the pathologist considers AMAG. However, histologic overlap likely exists between these two phenotypes depending on the clinical context.

Just as AMAG can present subtly both histologically and clinically, endoscopic findings can also be indistinct. This presents a quandary, because while AMAG must be confirmed by histology, the decision to perform a biopsy at certain locations is determined by endoscopic impressions. In our study, endoscopy reports were scored regarding conclusiveness for atrophy based on both the language of the report alone (ES1) and when the images were incorporated (ES2) by trained gastroenterologists. We found that ES1 and ES2 were highly correlated with each other, with a determination of “inconclusive gastritis” being the most common designation for patients. Endoscopic impressions (both ES1 and ES2) did not correlate with ECL cell hyperplasia, histologic atrophy grade, or histology comment score. Furthermore, our results suggest that endoscopists tend to use stronger terminologies that are relevant to AMAG when they intend to sample the antrum and corpus separately, or when a patient is female. On the other hand, pathologists’ interpretation of a biopsy appears to be independent of tissue sampling or the patient’s gender. Our overall findings correlate with a previous study, where, based on histology, the sensitivity of endoscopic impressions of gastric corpus atrophy was only 47% [8]. The authors also found that endoscopic diagnosis of atrophy decreased in accuracy when the patient was younger than 50 years old and when chronic inflammation was more severe. Chronic gastric inflammation may indeed reduce submucosal vascularity, obscuring macroscopic signs of atrophy to the endoscopist’s eye [21]. We speculate that under-recognition of the endoscopic features of AMAG is likely due to the nonspecific nature of the disease, rather than training or experience. AMAG can present with variable, non-specific findings, including flattening of rugal folds, thinned mucosa, polypoid lesions, or it may be indistinguishable from a healthy gastric corpus in the case of early-stage AMAG [22].

Our results demonstrate clear inconsistencies regarding AMAG patient follow-up testing. We observed that patients who presented with documented NET were closely followed with additional biopsies (10 of 12), while patients who presented with AMAG alone were not (14 of 53). Furthermore, there was no association between the histology comment score and whether the clinician performed a follow-up biopsy. However, patients who did receive follow-up biopsies tended to have greater atrophy on histology and significant ECL cell hyperplasia, indicating that the endoscopist’s judgment regarding the severity of endoscopic abnormality at the time of biopsy may have impacted follow-ups to a certain extent, regardless of the pathologist’s input. In contrast, when at least one relevant laboratory test was performed (in 88% of patients), the histology comment strength was positively associated with ordering three or more tests. This appears to indicate that a pathologist’s input plays a role when determining how many tests to order. This is dissimilar to another study conducted by practicing pathologists, wherein less than half of patients with an AMAG diagnosis based on histology received serologic follow-up testing [13]. This discrepancy may be because they evaluated a narrower range of tests (anti-parietal cell and anti-intrinsic factor antibodies, vitamin B12, and gastrin) while we evaluated seven tests. Hemoglobin is a common, routine laboratory test not specific to AMAG diagnosis, which may be why our follow-up serological test rate was so high. Nonetheless, the AGA recommends that when AMAG is suspected, clinicians should evaluate for anti-parietal cell and anti-intrinsic factor antibodies, *H. pylori*, and anemia due to iron and vitamin B12 deficiencies [12]. Ultimately, the diagnosis of AMAG relies on histopathological examination as the gold standard, with serological testing as a supporting element that also helps to inform on further patient management [12,13]. As previously mentioned, the endoscopic surveillance interval for AMAG patients is unclear, but it is recommended that endoscopy be performed at least every 3 years for patients with advanced atrophy [12].

The lack of follow-up overall in our study may be due to a variety of factors, but a clinical misunderstanding of AMAG could be a possibility. While it is true that there is currently no specific treatment for AMAG, it is a common cause of severe vitamin B12 deficiency, and supplementing patients can prevent the onset of neurological compromise [23]. Perhaps clinicians may be more familiar with the term “pernicious anemia”, which could be used in the pathology report to increase clarity when making comments on the implication of an AMAG diagnosis. Furthermore, explicitly recommending appropriate ancillary testing could be an additional measure to take. Ultimately, clear communication between the pathologist and gastroenterologist is important. AMAG can present asymptomatically and is often not clinically suspected, so in this case it lies in the hands of the pathologist to alert the clinician to this diagnosis.

Our findings highlight the need for the development of a comprehensive, standardized follow-up protocol for AMAG without NET. Since pathologists have had a clear understanding regarding diagnostic criteria for AMAG for decades [24], this improvement to patient care is certainly overdue. Our study accords with another recent retrospective study which demonstrated that this patient population is not re-biopsied as frequently as patients with type I gastric NETs [25]. The strengths of our study include its focus on current clinical practices, providing a practical observation and assessment of adherence to biopsy protocols and the influence of language used on pathology reports on subsequent testing. The inclusion of patients with and without NETs allowed for a comparative analysis of follow-up patterns, revealing disparities which may have significant clinical implications. The limitations include potential limiting of generalizability, as the study reflects the practices of a single institution. Secondly, this is a retrospective study, and thus, the parameters were not controlled. Prospective multicenter studies are needed to validate our findings and evaluate the effect of standardized biopsy protocols on both diagnostic accuracy and patient outcomes. Standardizing serological tests may be beneficial as well. For example, even though pepsinogen and gastrin have proved to be reliable markers of oxyntic mucosa atrophy, these functional serological tests are not included in international protocols [16]. Investigating enhanced endoscopic techniques, such as AI-assisted imaging or narrow-band imaging could be a method to improve endoscopic and histologic correlation in AMAG, which our study found to be poor. Longitudinal studies are essential to establish evidence-based surveillance protocols for patients with AMAG, to ensure early detection of malignant neoplasm.

## 5. Conclusions

In conclusion, this study underscores critical gaps in the diagnosis and management of AMAG, revealing that endoscopic impressions poorly correlated with histologic severity and that patients with AMAG without NET were less likely to receive follow-up biopsy regardless of the strength of language used in the pathology report. This inconsistency in surveillance practices emphasizes the need for standardized biopsy protocols and clear pathology reporting to ensure timely diagnosis and appropriate follow-up. We are hopeful that by addressing these deficiencies through guideline-driven practices and future research, clinicians can enhance patient care, reduce the risk of underdiagnosis, and improve clinical outcomes for AMAG patients.

## Figures and Tables

**Table 1:** Histology comment score.

	Histology Comment Score
1	Alternative differential diagnosis including early AMAG
2	Possible early AMAG
3	Can be seen in AMAG/atrophic gastritis, NOS
4	Suggestive of AMAG/features resembling AMAG
5	Consistent with/suspicious for AMAG

AMAG: autoimmune metaplastic atrophic gastritis; NOS: not otherwise specified.

**Table 2:** Endoscopy report score.

	Endoscopy Report Score
1	Normal gastric mucosa
2	Gastric erythema
3	Inconclusive gastritis (papules/nodules/pseudopolyps/mildly edematous mucosa)
4	Features of atrophy reported (mosaic pattern, nodular appearance, loss of gastric folds)
5	Atrophy present (visible blood vessels in corpus)

**Table 3:** Descriptive statistics of the study cohort.

**Demographics**	
Age (mean, median, range)	64.6, 65, (30–86)
Sex (*n*, %)	
Male	25 (38)
Female	40 (62)
**Clinical Findings**	
Other autoimmune conditions (*n*, %)	15 (23) (NA = 4) ^a^
Hypothyroidism	7 (11)
Hashimoto’s thyroiditis	1 (2)
Graves’ disease	1 (2)
Type 1 diabetes	2 (3)
Other	4 (7)
Indications for endoscopic evaluation (*n*, %)	
Anemia/GI bleeding	19 (29)
Dysphagia/pain	27 (42)
Follow-up for gastric pathology/neoplasm	18 (28)
Follow-up for esophageal pathology	8 (12)
Other GI related symptoms	8 (12)
Others	5 (8)
Biopsy sites (*n*, %)	
Antrum	31 (48)
Corpus	30 (46)
Cardia	4 (6)
Greater curvature	0 (0)
Lesser curvature	1 (2)
Random	21 (32)
Targeted biopsy	32 (49)
w/ flat mucosa evaluation	21 (32)
w/o flat mucosa evaluation	11 (17)
**Biopsy Review**	
Biopsy sites showing features of AMAG or NET (*n*, %)	
Antrum	2 (3)
Corpus	46 (71)
Cardia	5 (8)
Greater curvature	2 (3)
Lesser curvature	1 (2)
Random	19 (29)
Targeted biopsy	29 (45)
Targeted lesions	
Polyp/nodule/mass ^b^	20 (69)
Ulcer/erosion ^b^	6 (21)
Scar ^b^	2 (7)
Others ^b^	3 (10)
Targeted biopsy shows NET ^b^	10 (34)
Atrophy grade (mean, median, range)	2.8, 3, (1–3)
Synaptophysin and/or chromogranin IHC (*n*, %)	61 (94)
ECL hyperplasia pattern	
Negative	1 (2)
Linear	5 (8)
Linear and micronodular	47 (72)
NET	12 (18)
* Helicobacter pylori* IHC (*n*, %)	59 (91)
Positive ^b^	3 (5)
Negative ^b^	56 (95)
**Histology Comment Score**	
Histology comment score (mean, median, range)	4.2, 5, (1–5) (NA = 2)
1	3 (5%)
2	3 (5%)
3	11 (17%)
4	7 (11%)
5	39 (57%)
**Endoscopy Report Score**	
Endoscopy report score #1 (mean, median, range)	2.9, 3, (1–5) (NA = 2)
1	9 (14%)
2	14 (22%)
3	24 (37%)
4	7 (11%)
5	9 (14%)
Endoscopy report score #2 (mean, median, range)	3.1, 3, (1–5) (NA = 2)
1	5 (8%)
2	16 (25%)
3	20 (31%)
4	12 (18%)
5	10 (15%)
**Follow-Up**	
Follow-up available (*n*, %)	26 (40)
Follow-up duration (months; mean, median, range)	29.4, 26.3, (2–84)
Follow-up biopsy (*n*, %)	
Performed	24 (37)
Not performed	41 (63)
Number of follow-up biopsy (mean, median, range)	2.2, 2, (1–8)
Follow-up laboratory testing (*n*, %)	
Performed	57 (88)
Not performed	8 (12)
Number of follow-up laboratory testing (mean, median, range)	2.4, 2, (0–5)

w: with; w/o: without; GI: Gastrointestinal; AMAG: Autoimmune metaplastic atrophic gastritis; NET: Neuroendocrine tumor; ECL: Enterochromaffin-like; IHC: immunohistochemistry. ^a^ The number indicates total number of conditions in 13 patients. ^b^ Percentage values were obtained by subgroup count as the denominator.

**Table 4:** Correlation analysis of endoscopic and pathologic findings: *p* value adjusted by FDR.

Endoscopy report score #1	-	*p* < 0.01 * r_s_ = 0.78	*p* = 0.06 r_s_ = 0.30	*p* = 0.72 r_s_ = 0.09	*p* = 0.75 r_s_ = 0.04
Endoscopy report score #2	*p* < 0.01 * r_s_ = 0.78	-	*p* = 0.52 r_s_ = 0.14	*p* = 0.54 r_s_ = 0.13	*p* = 0.75 r_s_ = 0.05
ECL hyperplasia	*p* = 0.06 r_s_ = 0.30	*p* = 0.52 r_s_ = 0.14	-	*p* = 0.75 r_s_ = 0.05	*p* = 0.04 * r_s_ = 0.32
Histology comment score	*p* = 0.72 r_s_ = 0.09	*p* = 0.54 r_s_ = 0.13	*p* = 0.75 r_s_ = 0.05	-	*p* = 0.52 r_s_ = 0.15
Atrophy grade	*p* = 0.75 r_s_ = 0.04	*p* = 0.75 r_s_ = 0.05	*p* = 0.04 * r_s_ = 0.32	*p* = 0.52 r_s_ = 0.15	-
	Endoscopy report score #1	Endoscopy report score #2	ECL cell hyperplasia	Histology comment score	Atrophy grade

FDR: false-discovery rate; ECL: enterochromaffin-like; r_s_: Spearman’s rho; * *p* < 0.05.

**Table 5:** AMAG-related laboratory tests performed in study cohort.

Laboratory Test	Number (%) of Patients with Performed Test
*H. pylori* serology	2 (3.1)
Serum chloride	48 (73.8)
Anti-parietal cell antibody	12 (18.5)
Vitamin B12	29 (44.6)
Hemoglobin	54 (83.1)
Serum gastrin	13 (20.0)
Serum pepsinogen I/II	0 (0.0)

## Data Availability

The original contributions presented in this study are included in the article. Further inquiries can be directed to the corresponding authors.

## Abbreviations

The following abbreviations are used in this manuscript:

AMAG	Autoimmune metaplastic atrophic gastritis
NET	Neuroendocrine tumor
ECL	Enterochromaffin-like
HCl	Hydrochloric acid
OLGA	Operative Link for Gastritis Assessment
US	United States
H&E	Hematoxylin and eosin
GI	Gastrointestinal
ANA	Antinuclear antibody
EGD	Esophagogastroduodenoscopy
ES1	Initial endoscopy report score based on report terminology
ES2	Subsequent endoscopy report score based on endoscopy images
AGA	American Gastroenterological Association
ENETS	European Neuroendocrine Tumor Society
